# Pilot study: advanced haemodynamic monitoring after acute spinal cord injury-Keep the pressure up?

**DOI:** 10.1186/s12871-022-01806-2

**Published:** 2022-09-01

**Authors:** Niklas Drotleff, Oliver Jansen, Christina Weckwerth, Mirko Aach, Thomas Armin Schildhauer, Christian Waydhas, Uwe Hamsen

**Affiliations:** 1 Department of General and Trauma Surgery, BG University Hospital Bergmannsheil, Bochum, Germany; 2 Faculty of Psychology, FernUniversität Hagen, Hagen, Germany; 3 Department of Spinal Cord Injury, BG University Hospital Bergmannsheil, Bochum, Germany; 4 Medical Faculty of the University of Duisburg-Essen, Essen, Germany

**Keywords:** Spinal cord injury, Advanced haemodynamic monitoring, Transpulmonary thermodilution, MAP goals

## Abstract

**Background:**

Although the use of vasopressors to maintain haemodynamic goals after acute spinal cord injury (SCI) is still recommended, evidence regarding the target values and possible risks of this practice is limited, and data on haemodynamic parameters unaffected by catecholamines are rare. In this pilot study, we show the haemodynamic profile of patients with acute SCI mainly unaffected by vasopressor use and other factors that influence the cardiovascular system.

**Methods:**

From March 2018 to March 2020, we conducted a prospective, single-centre pilot study of 30 patients with acute SCI. Factors that could affect the cardiocirculatory system other than SCI (sepsis, pre-existing heart disease or multiple trauma) led to exclusion. A total of 417 measurements were performed using the PiCCO™ system.

**Results:**

The mean systemic vascular resistance index (SVRI, *1447.23* ± 324.71 dyn*s*cm^−5^*m^*2*^), mean central venous pressure (CVP, *10.69* ± *3.16*) and mean global end-diastolic volume index (GEDVI, 801.79 ± 158.95 ml/m^2^) deviated from the reference range, while the mean cardiac index (CI), mean stroke volume index (SVI), mean arterial pressure (MAP), and mean heart rate (HR) were within the reference range, as indicated in the literature. A mixed model analysis showed a significant negative relationship between norepinephrine treatment and MAP (83.97 vs. 73.69 mmHg, *p* < 0.001), SVRI (1463.40 vs. 1332.14 dyn*s*cm^−5^*m^2^, *p* = 0.001) and GEDVI (808.89 vs. 759.39 ml/m^2^, *p* = 0.001).

**Conclusion:**

These findings could lead to an adaptation of the target range for SVRI and MAP in patients with acute SCI and therefore reduce the use of vasopressors.

## Background

Acute spinal cord injury (SCI) can lead to haemodynamically relevant vasoplegia of the peripheral vessels and bradycardia due to the abrupt loss of sympathetic control and overstimulation of the parasympathetic nervous system. The extent of these symptoms depends on the level of the SCI, with lesions above Th6 being particularly associated with haemodynamic instability [1]. However, the loss of motor function in lesions below Th6 may also contribute to a loss of peripheral resistance due to blood stasis and oedema in the lower limbs. The resulting autonomic dysreflexia in both groups can lead to severe orthostatic hypotension, which conflicts with the need for early mobilization [2, 3]. In severe cases, SCI can lead to neurogenic shock in which organ perfusion is critically reduced [4–9]. Therefore, patients with acute SCI are considered haemodynamically unstable, and many initially need intensive care surveillance and treatment, including close haemodynamic monitoring and vasopressor therapy [10–12]. The PiCCO™ system is an established method for haemodynamic monitoring and is routinely used in haemodynamically unstable intensive care patients, including those with acute SCI [13–16].

There are limited data on cardiocirculatory responses in patients with acute SCI. While a few previous studies assessed haemodynamics using the Swan-Ganz catheter, the results obtained were always biased by vasopressor use and were limited to the first week after SCI [11, 17]. Our aim was to show the haemodynamic profile of patients suffering from acute SCI over a longer period of time and unaffected by vasopressor use as well as other factors that might influence the cardio circular system, i.e., heart failure and sepsis.

## Methods

### Study design and oversight

The study was conducted prospectively in a single centre. Written informed consent was obtained from the patients or their legal representatives prior to inclusion. The study was approved by the Ethics Committee of the Ruhr University Bochum (No. 17–6002-BR).

### Patient population

All patients treated in the intensive care unit (ICU) of the University Hospital Bergmannsheil Bochum between March 2018 and March 2020 suffering from traumatic SCI with an acute onset of neurological symptoms (< 24 h) and who required regular arterial blood gas analyses or invasive blood pressure monitoring as part of their treatment were eligible for inclusion. The patients underwent neurological examination according to the International Standards for Neurological Classification of Spinal Cord Injury (ISNCSCI) by the American Spinal Injury Association (ASIA) and the International Spinal Cord Society [18]. Patients younger than 18 years of age as well as patients who did not require regular blood gas analysis or invasive blood pressure monitoring were not included. Furthermore, patients with known heart failure were not included. In cases with unavailable heart failure information, we excluded all patients with a history of heart attack, cardiovascular intervention, including coronary stenting or bypass surgery, and cardiac arrhythmia. In addition, patients with elevated inflammatory parameters (CRP > 10 mg/dl and/or leucocytes > 13/nl) and/or a body temperature > 39 °C and patients receiving antibiotics were not included. If an included patient showed signs of inflammation or sepsis, then these measurements were not used for the analysis. Furthermore, patients with multiple injuries (injury severity score > 16) were excluded.

### Measurements

The “PICCO2” system from Pulsion (PiCCO™, PULSION Medical Systems, Munich, Germany) with a module for the M1046A monitor system from Philips was used to measure the cardiac index (CI), the stroke volume index (SVI), the systemic vascular resistance index (SVRI) and the global end-diastolic volume index (GEDVI). The measurements were performed according to the manufacturer’s manual (Instructions for Use Picco2, Version 3.0). As part of the standard intensive care monitoring, an arterial catheter and a central venous catheter (CVC) were placed. In all patients, the thermodilution solution was administered via a central venous catheter placed either in the internal jugular or subclavian vein. The choice of catheter was left to the attending physicians. A chest X-ray was performed to ensure the correct placement of the catheter tip. The arterial catheter (PiCCO catheter 5F 20 cm) was always placed in the femoral artery. In patients with SCI, placing a femoral arterial catheter is standard practice in our ICU, as it allows for hindrance-free training of the upper body. Furthermore, early mobilization can be achieved because catheter placement in a large arterial vessel ensures a continuous measurement even with a flexed hip. However, catheters placed in the radial, brachial or axillary artery are smaller and more likely to malfunction due to voluntary or involuntary movement of the arms. For the individual measurements, the application of 4 × 20 ml cold (8–10 °C) NaCl 0.9% solution was used. Saline solution was administered as recommended in the Instructions for Use of the PiCCO system, since glucose solutions have been associated with malfunction of the injection valve at the CVC. Furthermore, as thermodilution boluses accounted for at least 250 ml/day, this volume of 5% glucose solution might have affected blood sugar levels in diabetic and nondiabetic patients. During the ICU stay, the haemodynamic parameters were measured by means of thermodilution at least 3 times a day (once per shift in a three-shift system). Additionally, the heart rate (HR), MAP, central venous pressure (CVP) and use of vasopressors were noted. CVP measurement was performed according to the usual practices of our ICU. The pressure measurement device was connected to the distal lumen of the CVC. It was then positioned at the mid thoracic level. The zero reference was carefully calibrated. The mean CVP value displayed on the monitor was noted. In ventilated patients, the mean CVP was documented after three respiratory cycles without mechanical ventilation interruption. The only vasopressor administered was norepinephrine. The indication to start pharmacological vasoconstriction was decided by the attending physician. In general, pharmacological vasoconstriction was initiated when the MAP dropped below 70 mmHg or when the patient presented signs of reduced organ perfusion, i.e., elevated serum lactate, oliguria, and dizziness. As a standard practice in the ICU of the Bergmannsheil Bochum University Hospital, all catheters are removed at the earliest possible time regardless of the underlying disease. The measurements were stopped once regular arterial blood gas analyses or invasive blood pressure monitoring were no longer indicated. In acute SCI patients, measurements were stopped when the patients were haemodynamically stable in bed and during mobilization as well as when mechanical ventilation was no longer required or when patients requiring prolonged ventilation showed a stable blood gas analysis for more than 48 h.

### Statistical analysis

The data were stored in a strictly pseudonymous form in Excel (Microsoft® Excel® for Microsoft 365 MSO). The statistical evaluation was conducted with SPSS statistical software (Version 25, IBM Corp., Armonk, NY, USA) and R Core Team (Version 2017, R: A language and environment for statistical computing, R Foundation for Statistical Computing, Vienna, Austria). The data were first aggregated by subject, and then a descriptive statistical analysis (mean, standard deviation, standard error) was conducted. A mixed model analysis was performed to determine whether there were any differences in the values of the respective criterion variables during the administration of norepinephrine.

## Results

Thirty patients (age 55 ± 19 years, 27 males and 3 females) were included. In total, 417 measurements were performed. Twenty-two patients were mechanically ventilated on at least one measurement (278 measurements, *n* = 417). The highest PEEP was 10 cmH_2_O (mean PEEP 6.95 ± 1.876 cmH_2_O). Only 8 patients were sedated on at least one measurement (83 measurements, *n* = 417). On average, the first measurement was taken 12 ± 18 days after SCI. Data were collected over a period of 8 ± 5 days. Three of the patients died during their stay in the hospital after being released from the ICU (Table 1).

**Table 1:** Patient population and length of stay

	N	Minimum	Maximum	Mean ± SD
Age (years)	30	18	82	55 ± 19
Weight (kg)	30	60	88	77 ± 7
Height (cm)	30	160	190	179 ± 8
Body Surface Area (m^2^)	30	1.6	2.3	2.0 ± 0.1
Time to first Measurement (days)	30	1	89	12 ± 18
Mean duration of Measurement (days)	30	1	20	8 ± 5
Length of ICU stay (days)	30	6	49	22 ± 12
Length of hospital stay (days)	30	22	370	136 ± 95

*ICU* Intensive care unit, *SD* Standard deviation

Twenty-five subjects suffered from an injury to the cervical or high-thoracic spinal cord (C2-Th6). Five patients had a lesion of the lower thoracic or lumbar spine. Most patients suffered from a severe cervical or high-thoracic SCI according to the ASIA Impairment Scale (AIS). (Table 2).

**Table 2:** Level and severity of SCI according to the ISNCSCI

Level	ASIA impairment score	n (%)
C2-Th6	AIS A	14 (56)
	AIS B	4 (16)
	AIS C	6 (24)
	AIS D	1 (4)
	Total	25 (100)
below Th6	AIS A	3 (60)
	AIS B	1 (20)
	AIS E	1 (20)
	Total	5 (100)

*ASIA *American Spinal Injury Association,* AIS *Asia Impairment Scale,* ISNCSCI *International Standards for Neurological Classification of Spinal Cord Injury,* SCI Spinal cord injury*

In the comparison of measurements regardless of vasopressor use, SVRI (1447.23 ± 324.71 dyn*s*cm^−5^*m^2^, 95%-confidence interval (95%-CI) [1323.72, 1570.75]), CVP (10.69 ± 3.16 mmHg, 95%-CI [9.48, 11.90]) and GEDVI (801.79 ± 158.95 ml/m^2^, 95%-CI [741.24, 862.16] deviated from the reference ranges (SVRI 1700–2400 dyn*s*cm^−5^*m^2^, CVP 1–7 mmHg, GEDVI 680-800 ml/m^2^) specified in the literature (Table 3), while CI, SVI, HR and MAP were within the reference range.

**Table 3:** Comparison of the mean values to the reference range

	N	Minimum	Maximum	Mean ± SD	95%-CI	Reference Range
CI (l/min/m^2^)	30	2.43	5.78	4.14 ± 0.84	[3.83, 4.46]	3.0–5.0
SVI (ml/m^2^)	30	37.17	82.04	58.69 ± 10.93	[54.57, 62.89]	40–60
SVRI (dyn*s*cm^−5^*m^2^)	30	973.22	2538.25	**1447.23 ± 324.71**	[1323.72, 1570.75]	1700–2400
HR (bpm)	30	54.88	100.00	72.62 ± 12.62	[67.82, 77.42]	60–80
MAP (mmHg)	30	65.88	112.43	81.55 ± 10.45	[77.57, 85.53]	70–90
CVP (mmHg)	30	3.33	15.41	**10.69 ± 3.16**	[9.48, 11.90]	1–7
GEDVI (ml/m^2^)	30	503.45	1132.00	**801.79 ± 158.95**	[741.24, 862.16]	680–800

Relevant deviation highlighted in bold

*CI* Cardiac index, *CVP* Central venous pressure, *GEDVI* Global end-diastolic volume index, *HR* Heart rate, *MAP* Mean arterial pressure, *SD* Standard deviation, *SVI* Stroke volume index, *SVRI* Systemic vascular resistance index, *95%-CI* 95%-confidence interval

Nineteen patients required vasopressor therapy with norepinephrine during their stay in the ICU on at least one day of measurements. To determine whether there were differences in the values of the respective criterion variables CI, SVI, SVRI, GEDVI, MAP and CVP during the administration of norepinephrine, a mixed model analysis was performed. All the criterion variables varied at the population level. The addition of the binary predictor variable “norepinephrine” showed a significant negative relationship with the SVRI, GEDVI and MAP (Table 4).

**Table 4:** Model parameters detecting differences in CI, SVI, SVRI, GEDVI, MAP and CVP with norepinephrine use

Parameter	Intercept only	Fixed slopes	*P*
*Regression coefficients (fixed effects)*			
Intercept (CI)	4.18	4.21	
Norepinephrin	-	-0.17	0.06
Intercept (SVI)	58.41	58.90	
Norepinephrin	-	-2.06	0.066
Intercept (SVRI)	1432.98	1463.40	
Norepinephrin	-	-131.26	0.001**
Intercept (GEDVI)	797.39	808.89	
Norepinephrin	-	-49.50	0.001**
Intercept (MAP)	81.59	83.97	
Norepinephrin	-	-10.28	< .001***
Intercept (CVP)	10.84	10.90	
Norepinephrin	-	-0.25	0.707
*Variance components (random effects)*			
Residuals/Intercept (CI)	0.48/0.61	0.48/0.63	
Residuals/Intercept (SVI)	71.03/105.74	70.39/106.17	
Residuals/Intercept (SVRI)	96536/83336	94204/81045	
Residuals/Intercept (GEDVI)	10367/22248	9968/23418	
Residuals/Intercept (MAP)	143.8/91.1	128.7/85.6	
Residuals/Intercept (CVP)	27.30/6.05	27.27/6.14	
*Intraclass Correlation Coefficients*			
CI	0.56		
SVI	0.59		
SVRI	0.46		
GEDVI	0.68		
MAP	0.38		
CVP	0.18		

Relevant deviation highlighted in bold

*CI* Cardiac index, *CVP* Central venous pressure, *GEDVI* Global end-diastolic volume index, *HR* Heart rate, *MAP* Mean arterial pressure, *SD* Standard deviation, *SVI* Stroke volume index, *SVRI* Systemic vascular resistance index

^**^p < .01

^***^p < .001

## Discussion

In this study, we performed invasive haemodynamic monitoring in patients suffering from acute SCI using the thermodilution-based PiCCO™ system. To our knowledge, this is the first study on advanced haemodynamic monitoring in patients with SCI who are not typically treated with vasopressors, and the first study to present data that are not biased by septic or cardiogenic shock.

Few studies have examined advanced haemodynamic parameters in patients with acute SCI. In 1993, Levi et al. performed haemodynamic monitoring via a Swan-Ganz catheter in 50 patients with SCI to maintain a haemodynamic profile with adequate cardiac output [17]. Similarly, Vale et al. maintained a mean arterial blood pressure above 85 mmHg in 77 patients with acute SCI and used a Swan-Ganz catheter to monitor the haemodynamic status [11]. Both prospective studies aimed to show that maintaining a certain haemodynamic profile improved the neurological outcome of patients with acute SCI and ultimately led to the recommendation to keep the MAP > 85 mmHg for the first 7 days after acute SCI. However, data were collected only within the first week after injury, and most measurements were obtained under the influence of vasopressors. Furthermore, the MAP goal and the period of time for that goal were selected arbitrarily. Another study used impedance cardiography to assess the haemodynamic profiles in 9 patients with neurogenic shock after acute SCI, but only one measurement was obtained from each patient on admission, and the use of vasopressors was not reported [19]. In 2019, Squair et al. monitored MAP and central spinal fluid pressure during the first week after injury and suggested that spinal cord perfusion pressure rather than MAP was an indicator of neurologic outcome [20]. As in previous studies, the measurements were taken within the first week postinjury, and vasopressors were used to maintain the MAP goals.

In contrast, our data consist of haemodynamic measurements mostly obtained without the influence of vasopressors. Factors that could affect the cardiocirculatory system other than SCI, such as infections, sepsis or pre-existing heart disease, led to the exclusion of some patients. Hence, our study provides a mostly unbiased haemodynamic profile of patients with acute SCI.

SVRI was reduced significantly compared to the resistance considered normal in non-SCI patients regardless of whether they required vasopressor therapy. Since the SVRI is calculated from the MAP, the CVP and the CI, the reduced vascular resistance can be attributed to a change in one of these values. A consistently high CVP, a high-normal CI and a standard MAP were shown with catecholamine therapy and without pharmacological vasoconstriction. Our data therefore suggest that in acute SCI, cardiac output is increased to compensate for the loss of the afterload (SVRI). The increase in cardiac output could be explained by an increase in preload. The CVP is used as a marker for preload in the formula for SVRI mentioned above. Since venous pressure can be influenced by many factors and is not recommended on its own for either volume status or fluid responsiveness [21], we included the GEDVI as a preload marker. In accordance with the CVP, the GEDVI lies at the upper margin of the reference range. This supports the theory that the cardiac output is increased by an augmented preload. All patients’ volume status was closely monitored with the thermodilution method, and the fluid balance was corrected according to the volumetric measurements to achieve euvolemia. However, an artificially elevated preload due to a positive fluid balance might also contribute to an increase in the cardiac output. Since our data do not show a decrease in the cardiac index usually associated with hypervolemia, however, the fluid balance and therefore the volume status must have been within a physiological range. In addition to a reduced SVRI independent of vasopressor use, the mixed model analysis shows a negative correlation between MAP, SVRI and GEDVI and norepinephrine treatment. CI, SVI and CVP were not affected by vasopressor use. As a standard practice in the ICU of the university hospital Bergmannsheil Bochum, a MAP of 70 mmHg is tolerated in patients with SCI if there are no signs of reduced organ perfusion, i.e., serum lactate, oliguria, or dizziness. Therefore, whenever the MAP dropped below 70 mmHg, norepinephrine was used to achieve a MAP of 70 mmHg. Hence, the MAP under vasopressor therapy (73.69 mmHg) was lower than that without norepinephrine treatment (83.97 mmHg) (Table 4). Since the calculation of the SVRI relies on the MAP, a negative correlation for the peripheral resistance due to the MAP goal was to be expected. Whenever vasopressor support was indicated, the loss of peripheral resistance could not be compensated for by an increase in preload and consecutively by an increase in the cardiac index. As a result, the GEDVI was also lower under norepinephrine treatment. Due to this compensation mechanism, sufficient perfusion seems to be maintained even with reduced peripheral resistance. From a cardiocirculatory point of view, it can be assumed that a lower resistance can be tolerated, and thus, an adjustment of the reference value is to be considered if the patient does not show signs of reduced organ perfusion.

In clinical practice, the aforementioned evidence led to relatively high MAP goals in patients with SCI. However, the resulting use of vasopressors and the consecutive need for invasive monitoring leads to delays in patient mobilization and longer hospitalization [22].

Our data suggest that adjustment of the target values for MAP and SVRI could be justified and could lead to a more focused application as well as reduced dosage of vasopressors. We acknowledge that this study did not assess the neurological outcome of patients and therefore cannot determine whether a more restrictive use of vasopressors for blood pressure management might lead to a worse neurological outcome. However, to our knowledge, there is no evidence that “high normal” blood pressure (MAP 85 mmHg) leads to a better neurological outcome than normotension. Rather, based on our experience with severe cerebral trauma, the spinal perfusion pressure seems to be responsible for a difference in outcome [20]. Therefore, a general recommendation of a “high normal” MAP goal in light of the aforementioned negative effects of vasopressor use should be critically questioned.

Furthermore, the use of the thermodilution-based PiCCO™ system could serve as a safe and easy-to-use means of haemodynamic monitoring in acute SCI. However, the use of thermodilution-based measurements is limited to the ICU and reserved for only those patients who need invasive blood pressure monitoring or regular blood gas analysis as part of intensive care treatment. The indication to maintain or remove a central catheter in the ICU setting, especially in the case of invasive blood pressure monitoring using vasopressors, must be questioned daily. In the author’s view, continuous surveillance of blood pressure in patients with SCI is a key aspect in early mobilization, as it allows for a quick and accurate adaptation of the vasopressor dosage if blood pressure suddenly drops. In the author’s experience, the arterial catheter itself does not delay early mobilization; rather, vasopressor therapy often hinders the physician from initiating mobilization. Especially in patients with acute SCI, blood pressure may vary greatly depending on the patient’s position. Therefore, in our view, invasive monitoring is mandatory in early mobilization.

As maintaining central catheters only for research purposes might escalate treatments without any clinical need, we did not specify a period of time for measurements in our study protocol. Therefore, the duration of measurements varies greatly between patients (Table 1). Additionally, the start of measurements was delayed depending on the duration of the initial external treatment (Table 1). Since the University Hospital Bergmannsheil Bochum is listed as a specialized centre for SCI, patients are admitted directly after trauma and transferred from various regions of Germany. Both adaptations to the study protocol render our study less comparable to the previously mentioned studies. However, the main focus of our study, i.e., assessment of haemodynamic profiles over a longer period of time, justifies this adaptation.

Some of the haemodynamic measurements were performed while patients were ventilated and/or sedated. As the haemodynamic profiles of a sedated and intubated patient could differ from a fully aware and spontaneously breathing one, ventilation and/or sedation may have biased the measurements. Furthermore, we did not differentiate between the neurological level/location of the SCI.

In addition, the number of patients enrolled in the study was very small. With the beginning of the COVID-19 crisis, the number of traumatic SCI cases dropped significantly, and fewer patients than initially anticipated were included. This could reduce the statistical power of our findings.

Further studies that examine the haemodynamic profiles of a larger sample of patients after acute SCI are needed for a better understanding of the cardiocirculatory changes caused by SCI. Long-term follow-up and regular assessment of the neurological status of these patients could show whether a certain haemodynamic profile might yield a higher or lower chance of neurological improvement and complications.

## Conclusions

In a cohort of patients with acute SCI, the systemic vascular resistance index was lower than the reference range. This was observed independent of the use of vasopressors. Cardiocirculatory monitoring using the PiCCO™ system is easy to use and could help define new cardiocirculatory goals in acute SCI.

## Data Availability

The datasets used and analysed during the current study are available from the corresponding author on reasonable request.

## Abbreviations

95% CI: 95% Confidence interval
AIS: ASIA Impairment Scale
ASIA: American Spinal Injury Association
CI: Cardiac index
CVC: Central venous catheter
CVP: Central venous pressure
DGUV: German Statutory Accident Insurance
GEDVI: Global end-diastolic volume index
HR: Heart rate
ICU: Intensive care unit
ISNCSCI: International Standards for Neurological Classification of Spinal Cord Injury
MAP: Mean arterial pressure
SCI: Spinal cord injury
SD: Standard deviation
SVI: Stroke volume index
SVRI: Systemic vascular resistance index
